# 3D finite element analysis of removable partial denture with lingual bar major connector constructed with cobalt chromium, PEEK, PEKK, and BioHPP

**DOI:** 10.1186/s12903-025-07493-y

**Published:** 2025-12-30

**Authors:** Aisha Zakaria Hashem Mostafa, Ahmed Sameh, Nermeen El sayed El-Khamisy

**Affiliations:** 1 Prosthodontic Department, Faculty of Dentistry, Mansoura University, Mansoura, Egypt; 2 Prosthodontic department, Faculty of Dentistry, Mansoura National University, Gamasa city, Egypt; 3 Production Engineering and Mechanical Design Department, Faculty of Engineering, Mansoura University, Mansoura, Egypt; 4 Mechatronics Department, Faculty of Engineering, Horus University, New Damietta, Egypt

**Keywords:** Von-Mises stress, Deflection, Major connector, Cobalt-chromium, RPD, Lingual bar, Polyetheretherketone (PEEK), Rigidity, Polyetherketoneketone (PEKK), Bio HPP

## Abstract

**Objective:**

The current study used finite element analysis (FEA) to investigate the rigidity, through Von-Mises stresses and deformation of cobalt-chromium, Polyetheretherketone (PEEK), Polyetherketoneketone (PEKK), and BioHPP constructed for mandibular class I RPD with lingual bar major connector. Although the general stress–strain relationships followed expected mechanical behavior, this study provides quantitative insight into how different polymeric materials (PEEK, PEKK, BioHPP) compare with conventional cobalt–chromium frameworks under identical loading conditions.

**Materials and methods:**

Four virtual models for mandibular Kennedy’s class I situations, with the remaining eight anterior and premolar teeth, were generated that closely resemble the actual cases. Mandibular cobalt-chromium class I RPD with lingual bar major connectors, half pear-shaped, with standard dimensions (50 × 5 × 3 mm), was constructed as a control group. Another 3 models similar to the control group in dimension but with 3 materials, PEEK, PEKK, and Bio HPP, were constructed as study groups. 3D finite element analysis was done, and data were collected and analyzed using bar chart.

**Results:**

The PEEK, PEKK, and Bio HPP major connectors showed more deformation (0.1798, 0.17554, 0.17104)mm, indicating weaker rigidity than the cobalt-chromium (0.028999)mm. The maximum von Mises stresses of the cobalt chromium lingual bar were 646.14 MPa, demonstrating stronger rigidity than the PEEK, PEKK, and Bio HPP, which exhibited values of 303.28, 295.29, and 301.23 MPa, respectively. In the case of PEEK and Bio HPP, greater stresses were transmitted to the supporting structures, particularly the mucosa, with maximum von Mises stress values of 3.0319 and 2.3047 MPa. These values were higher than the maximum stress transmitted to the mucosa in cobalt chromium, which was 0.60382 MPa. However, PEKK transmitted lower stresses to the supporting structures, particularly the mucosa, with a maximum von Mises stress value of 0.082096 MPa.

**Conclusion:**

Using the same size of PEEK, Bio HPP as the optimal dimensions of the cobalt chromium major connector of the mandibular class I RPD causes increased stress and deflection to the supporting structure, teeth, and soft tissues. This can be assigned to the PEEK’s, Bio HPP, lower stiffness compared to cobalt chromium. On the other hand, PEKK showed decreased stresses and deflection of the supporting structure.

When using PEEK and Bio HPP for class I RPD, the mandibular major connector must be larger.

**Supplementary Information:**

The online version contains supplementary material available at 10.1186/s12903-025-07493-y.

## Introduction

The primary goal of tooth-mucosa supported removable partial dentures (RPDs) is to replace lost teeth and related tissues with prosthetics without overstressing the remaining tissues [1, 2]. However, the two supporting structures, the teeth and the edentulous ridges, functionally distinct structures, cause axial and rotational stresses on the terminal abutment when the denture moves during mastication [3, 4]. Therefore, the primary challenge for any prosthodontist is to create a prosthesis that employs both support systems without subjecting the tissues to excessive stress [5, 6]. The implants can be positioned to assist a removable or fixed implant prosthesis [4]. However, the implant therapy alternative might not be practicable for several reasons; RPDs remain a viable and acceptable treatment option [7, 8]. The most common material used to manufacture RPD frameworks remains cobalt-chromium (Co-Cr) metal alloys due to their high elastic limit, superior resistance to wear and corrosion, biocompatibility, and affordability [9–11]. Although cobalt chromium has several benefits, including strength and low weight, the casting process is time-consuming and must be performed carefully to minimize human errors. Additionally, the use of metal clasps in these frameworks could occasionally be viewed as a drawback, particularly for patients who have high aesthetic demands. Digital manufacturing in dentistry has led to the development of novel techniques for creating cobalt-chromium frameworks. Furthermore, while creating high-performance polymers for use as frameworks for removable partial dentures has many benefits, it also raises questions about design principles and rules [12–15].

Non-metallic substances, such as polyether ether ketone (PEEK), have been developed for aesthetic and biocompatibility reasons [14, 16]. PEEK is an organic polymer that is semi-crystalline and has excellent mechanical and chemical qualities [17, 18]. PEEK is utilized in prosthetic treatments as an alternative for individuals who are allergic to metal because of its high heat resistance, its low weight, and biocompatibility also contribute to improved patient comfort and satisfaction [18–21]. To increase its strength, PEEK material can be mixed with fiber and ceramic materials [22, 23]. While PEEK has made it possible to fabricate RPDs that are more aesthetically pleasing than metal RPDs, its suitability has received less attention overall, particularly regarding the biomechanical requirements that are essential for the rigidity of the major connector [24].

There are modifications of PEEK, such as polyetherketoneketone (PEKK). The second ketone group in PEKK ensures better mechanical and physical properties [14]. PEKK has a higher compressive strength, excellent polishing ability, and a bone-like elastic modulus [24]. PEKK has been used for different types of prostheses [24–26]. A modified PEEK material containing 20% ceramic fillers is a high-performance polymer (BioHPP; Biocompatible high performance polymer), which presents high biocompatibility, good mechanical properties, high temperature resistance, and chemical stability. BioHPP is rigid, biocompatible, non-allergenic, and has flexibility like bone. It also has low plaque affinity, high polishing, low absorption, and strong wear resistance. It has been utilized for many years in prosthodontics [27–30].

The major connector is the unit of the RPD to which all other parts are directly or indirectly attached. It connects the parts on one side of the arch to those on the opposite side. The lingual bar is the most used mandibular major connector. The most important criteria of the major connector is the need to be rigid [25]. The major connector must be rigid to withstand bending, deformation, flexing, and torquing, which can cause damage to abutment teeth and other structures. The major connector is thus the most essential component, significantly subjected to maximum stress concentration and deflection due to many forces acting on it. Lingual bar major connector in Kennedy’s Class I cases showed the most deflection when compared to similar cases with lingual plate major connector [25, 26].

A partial denture’s main biomechanical function is to transfer constant forces to the teeth and the hard and soft tissues underneath [31]. Mechanical stress analysis, photoelasticity, and stereo photogrammetry are some methods for evaluating deflection. Over the last two decades, finite element analysis has been increasingly helpful [27]– [28]. The application of load to the prosthesis causes deflection. The magnitude and direction of the prosthesis’s deflection are determined by the rigidity of the major connector [29].

The finite element approach (FEA) is a valuable tool for predicting stress and displacement around dental materials. It shows how materials or methods behave in clinically simulated settings [32]. Also, it is highly effective in research [33]. Additionally, it is a computational modeling technique that has become integral in dental research, particularly in prosthodontics. It enables the assessment of complex interactions between dental prostheses and oral tissues under simulated physiological loading conditions. FEA divides a model into discrete elements to calculate stress, strain, and deformation across various components, making it highly suitable for evaluating the biomechanical behavior of removable partial dentures (RPDs), especially in Kennedy Class I scenarios where tissue support is critical [34]. In prosthodontics, FEA has been used to investigate the effects of connector designs, clasp types, materials, and implant placements on stress distribution and denture performance. Studies have shown that FEA results correlate well with physical measurements obtained through strain gauges and Photoelastic methods, validating its effectiveness as a predictive tool [35, 36].

PEEK, PEKK, and BioHPP have sufficient mechanical properties for use in removable partial denture frameworks, but Co-Cr alloys display better mechanical properties [37]. Design optimization is key to maximizing PEEK’s potential. Peng et al., 2020 [38], found that the use of PEEK for clasps performs comparably to Co-Cr in terms of retention. Previous studies have shown that the use of a PEEK mandibular lingual plate major connector increases the stresses on the mucosa [16]. Although the lingual bar of PEEK was used [19], no previous study has evaluated this lingual bar. So, this study aimed to investigate the rigidity, through Von-Mises stresses and deformation, of the lingual bar major connector made from PEEK, PEKK, and Bio HPP, and to compare them with that of cobalt chromium (Co-Cr) alloy by FEA.

The null hypothesis of this study is that there is no variation in stresses transferred to the supporting structures of the RPD and there will be no differences in the deformation of the lingual bar major connector when it made from cobalt chromium, PEEK, PEKK, and Bio HPP of the RPD in Kennedy’s Class I cases.

## Materials and methods

### Study design overview

This study utilized three-dimensional finite element analysis (FEA) to evaluate and compare the biomechanical performance of various materials, such as cobalt-chromium (Co-Cr) alloy and polyetheretherketone (PEEK), PEKK, and Bio HPP, in the same dimension, when used as lingual bar major connectors in mandibular Class I removable partial dentures (RPDs). The objective was to assess and compare stress distribution and deformation patterns in the prosthetic metal framework, including the lingual bar and supporting tissues, under simulated physiological loading.

### Anatomical modeling and framework design

A 3D digital model of the partially edentulous mandible, including the residual ridge, supporting mucosa, alveolar bone, and remaining dentition, including anterior teeth and first premolars, was constructed by using SolidWorks (Dassault Systems, France). The lingual bar was constructed with a dimension of 50 × 5 × 3 mm [5] in Co-Cr (control), PEEK, PEKK, and BioHPP frameworks (study).

These frameworks were digitally modeled and integrated into the mandibular arch using CAD software (SolidWorks© 2023). The frameworks were designed symmetrically to include, in addition to the mandibular lingual bar major connector, RPA clasp system (mesial rest, proximal plate, and Aker retentive arm) with engagement of the undercuts of the first premolars via this clasp and an indirect retainer in the form of a cingulum rest on the canine, ensuring clinical realism in stress transmission paths. The current FEA was designed to simulate a clinical situation of a partially edentulous mandible rehabilitated with a removable partial denture. A computer-aided design (CAD) 3D solid model was created based on real dimensions, followed by the application of the proposed material properties for each part to initiate the analysis process.

**Fig. 1:**
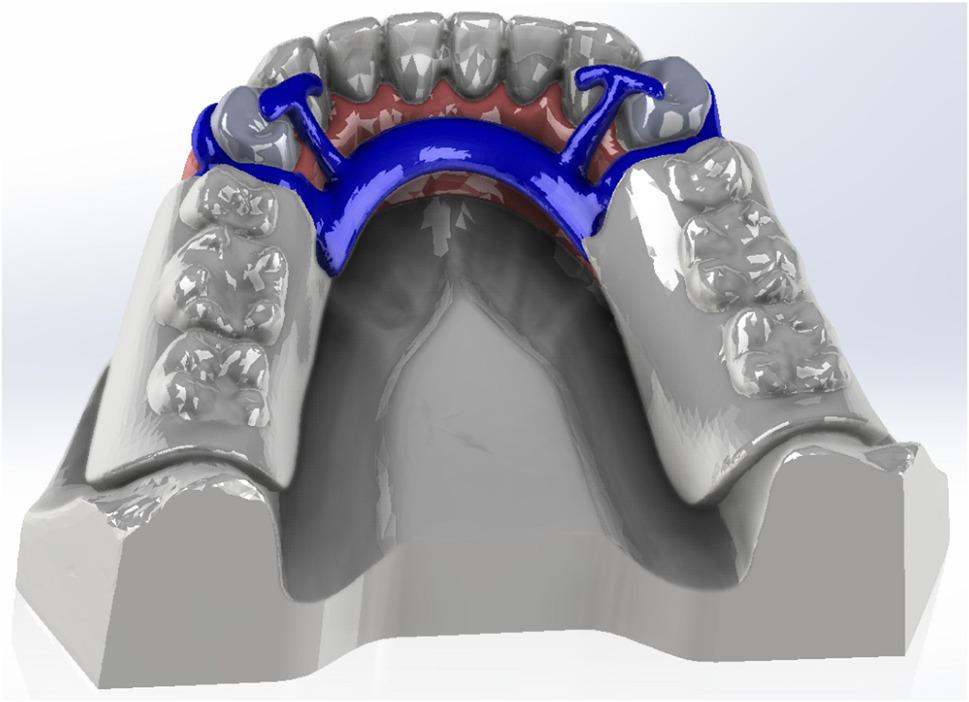
3D CAD modeling using SolidWorks

The proposed 3D virtual CAD model of the partially edentulous jaw (2.2 cm height × 1.8 cm width × 13.5 cm length) was established as shown in Fig. 1. The geometry was modeled using CAD software (SolidWorks© 2023; Dassault Systems SolidWorks Corp.) [39, 40].

### Material properties

The framework materials were assigned linearly elastic, isotropic, and homogeneous properties based on validated literature data (Table 1). All materials were assumed homogeneous, isotropic, and linearly elastic to allow a controlled comparison of the influence of elastic modulus on stress and deformation. This simplification is consistent with previously published finite element analyses of RPD frameworks [17, 38, 41]. Nonlinear and viscoelastic material behaviors were not modeled due to computational limitations and to maintain comparability among materials.

**Table 1.**
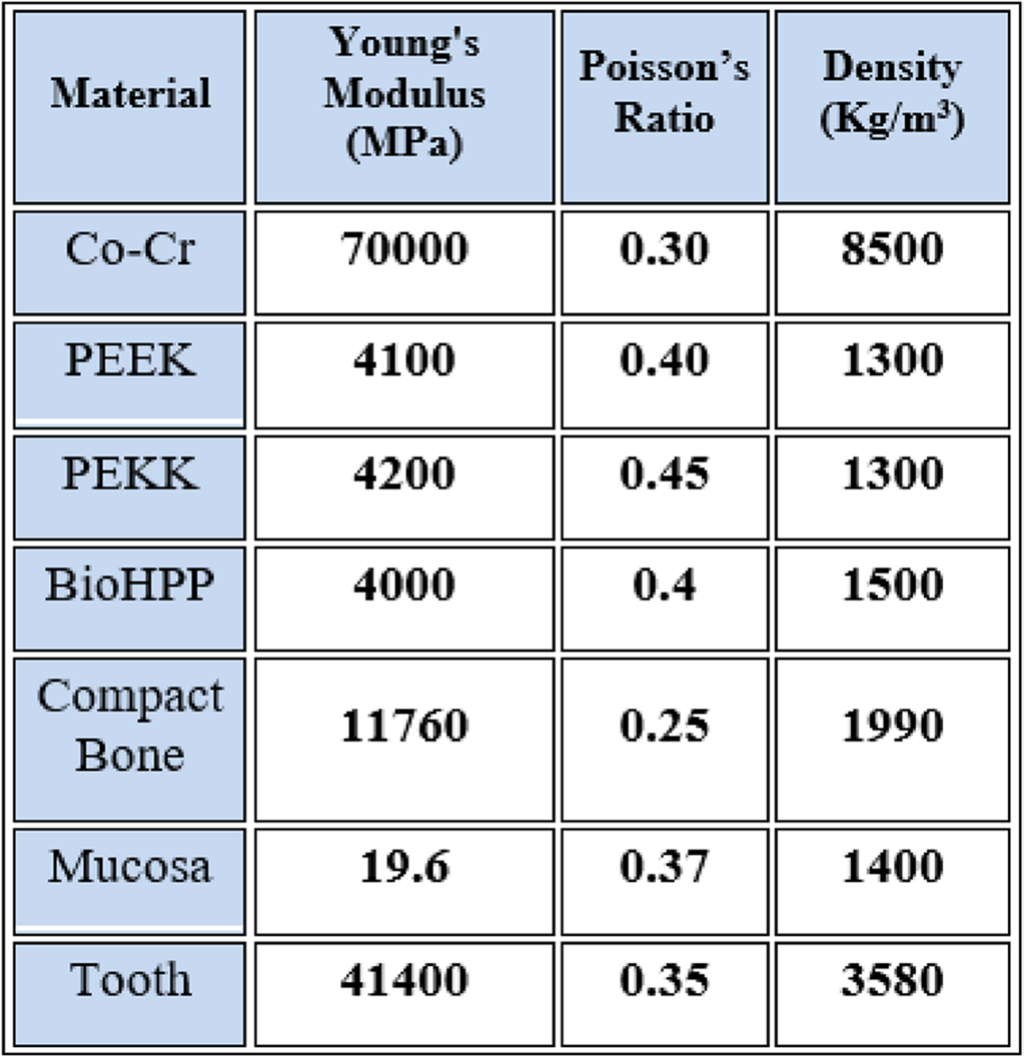
The mechanical properties of the materials[42–47]

### Finite-element mesh and convergence

The 3D assemblies were discretized using 10-node quadratic tetrahedral elements (ANSYS SOLID187). A mesh-refinement study was performed to some parts by sequentially decreasing element size from 1.5 mm → 1.0 mm → 0.6 mm. The model was considered converged when further refinement produced less than 3% variation in peak von Mises stress and total deformation. Tetrahedral elements with mesh refinement in stress-prone regions (frameworks and bone-tooth interfaces) ensured solution accuracy. The final mesh contained approximately 1.2 million elements, achieving convergence between medium and fine levels. Convergence and numerical stability were verified following standard procedures recommended in dental biomechanics FEA literature [17, 38].

### Model validation

Direct experimental validation was not included in the present computational phase. Instead, model reliability was ensured by reproducing boundary and loading conditions consistent with previously validated FEA models of removable partial dentures [17, 38]. The predicted stress distribution patterns were also cross verified against published experimental trends in the literature, showing comparable magnitudes and distribution behavior. Future research will include in vitro validation using 3D-printed frameworks and strain gauge measurements to further verify computational accuracy.

### Loading conditions

The inferior border of the mandible was fixed in all directions. A linear static analysis was performed under occlusal loads of 200 N (vertical) and 150 N (oblique), as previously reported by Nakamura et al. [48] and Jantaban N et al., [38]. This loading approach was chosen to allow direct comparison of the four framework materials under standardized geometry and boundary conditions. Nonlinear and cyclic loading analyses were not conducted due to computational constraints and to maintain methodological consistency with comparable published studies [17, 38].

### Analysis outputs

**Fig. 2:**
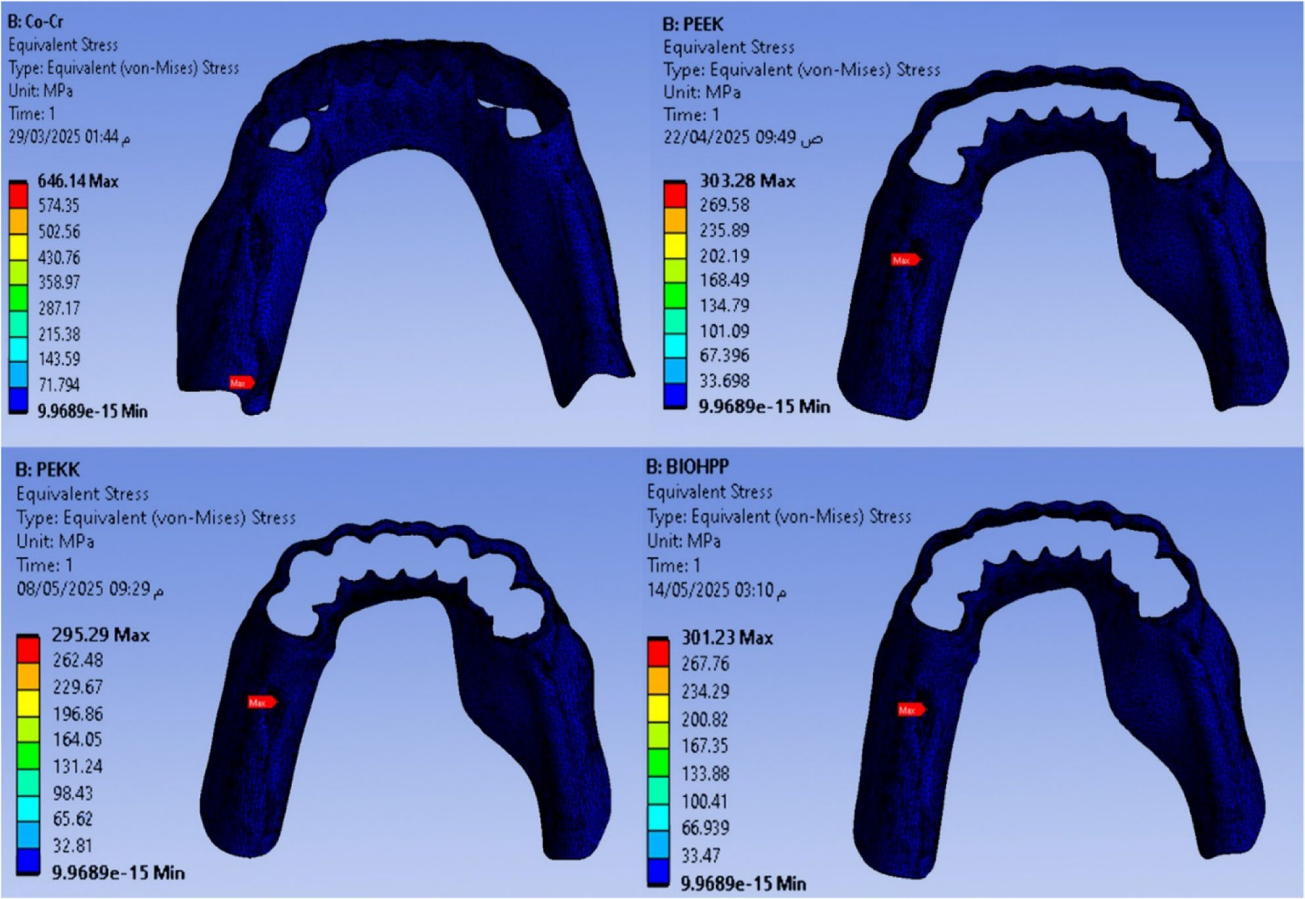
Von misses stress simulation in mucosa

**Fig. 3:**
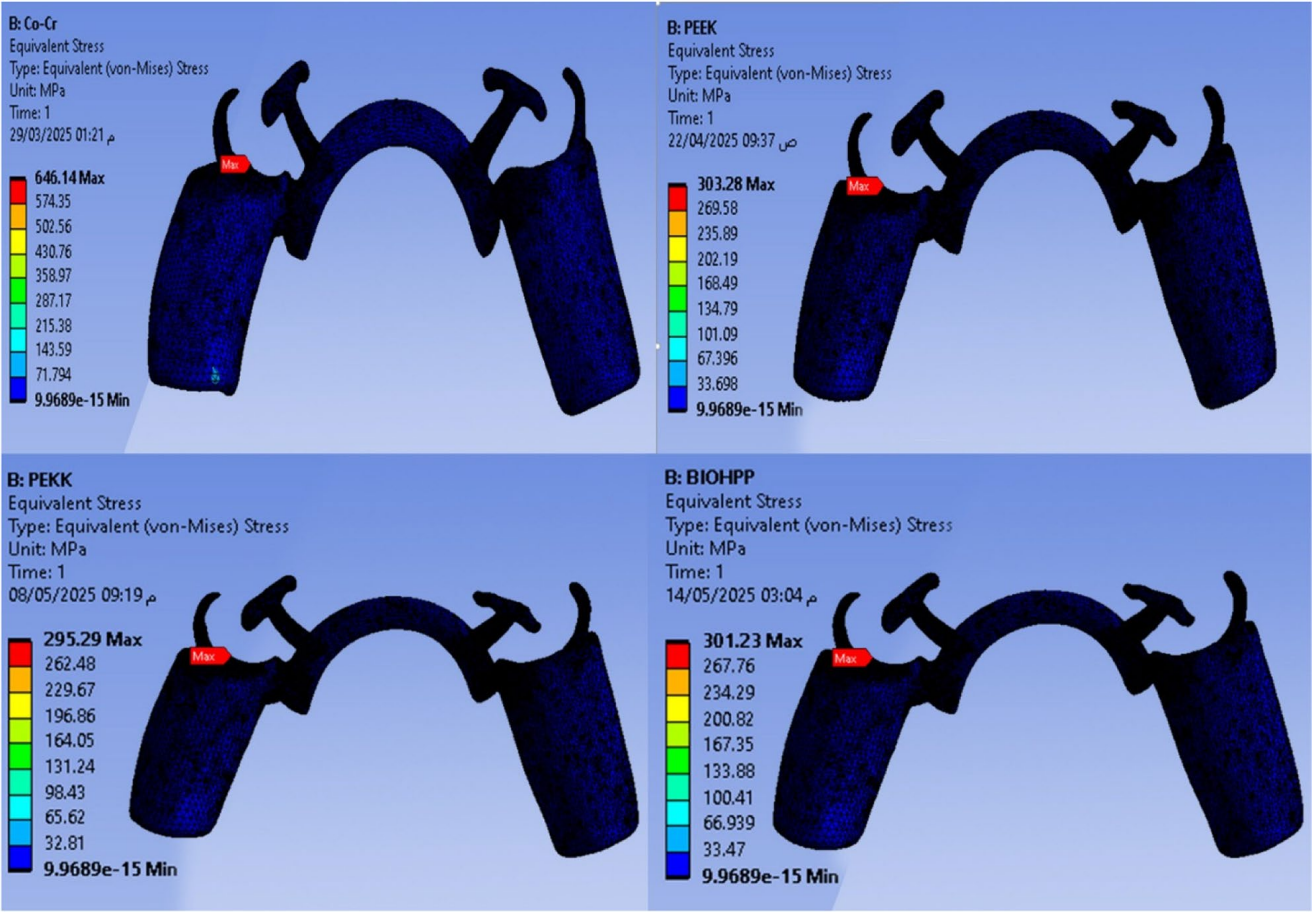
Von misses stress simulation in metal connector

**Fig. 4:**
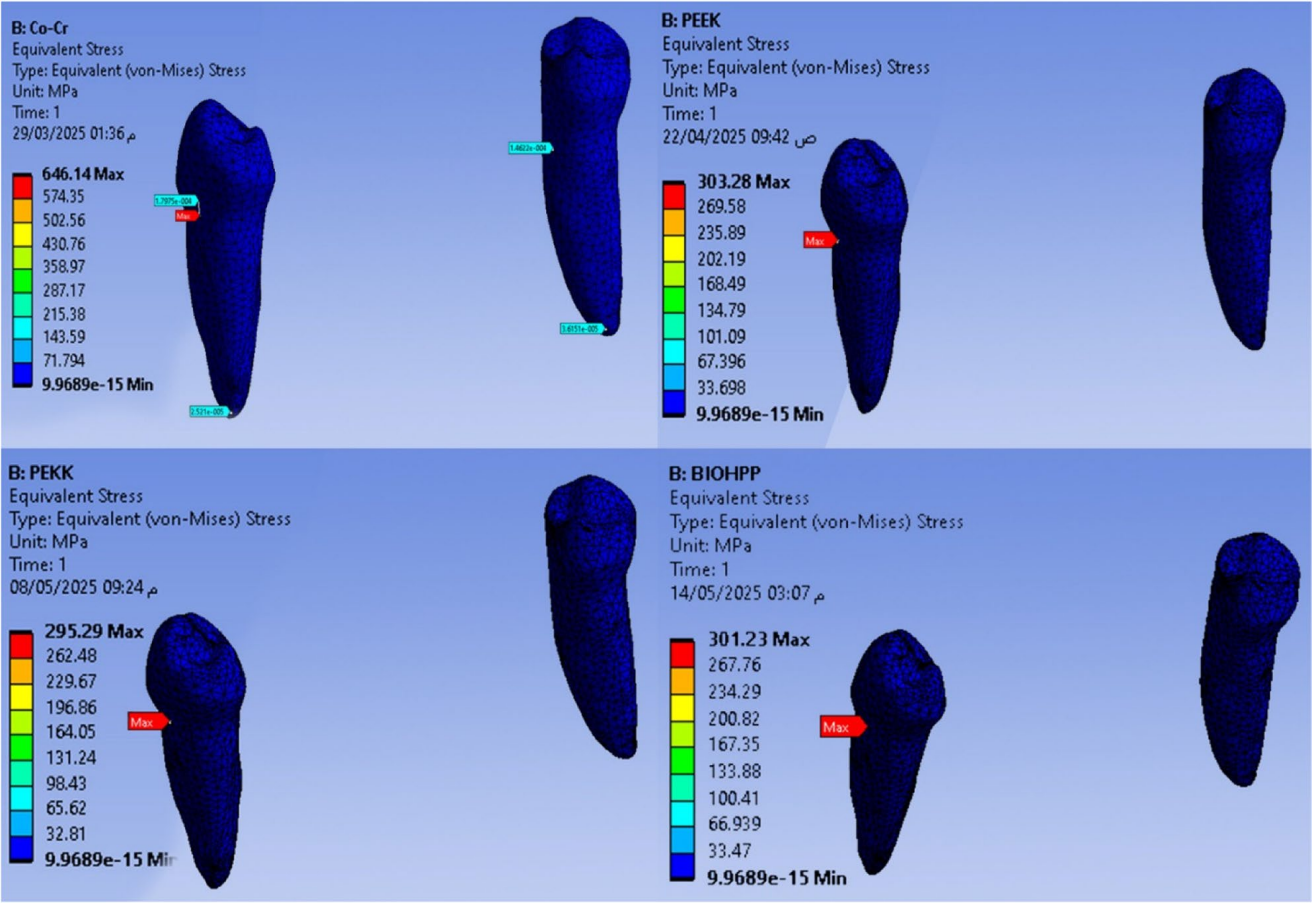
Von misses stress simulation in abutment teeth

**Fig. 5:**
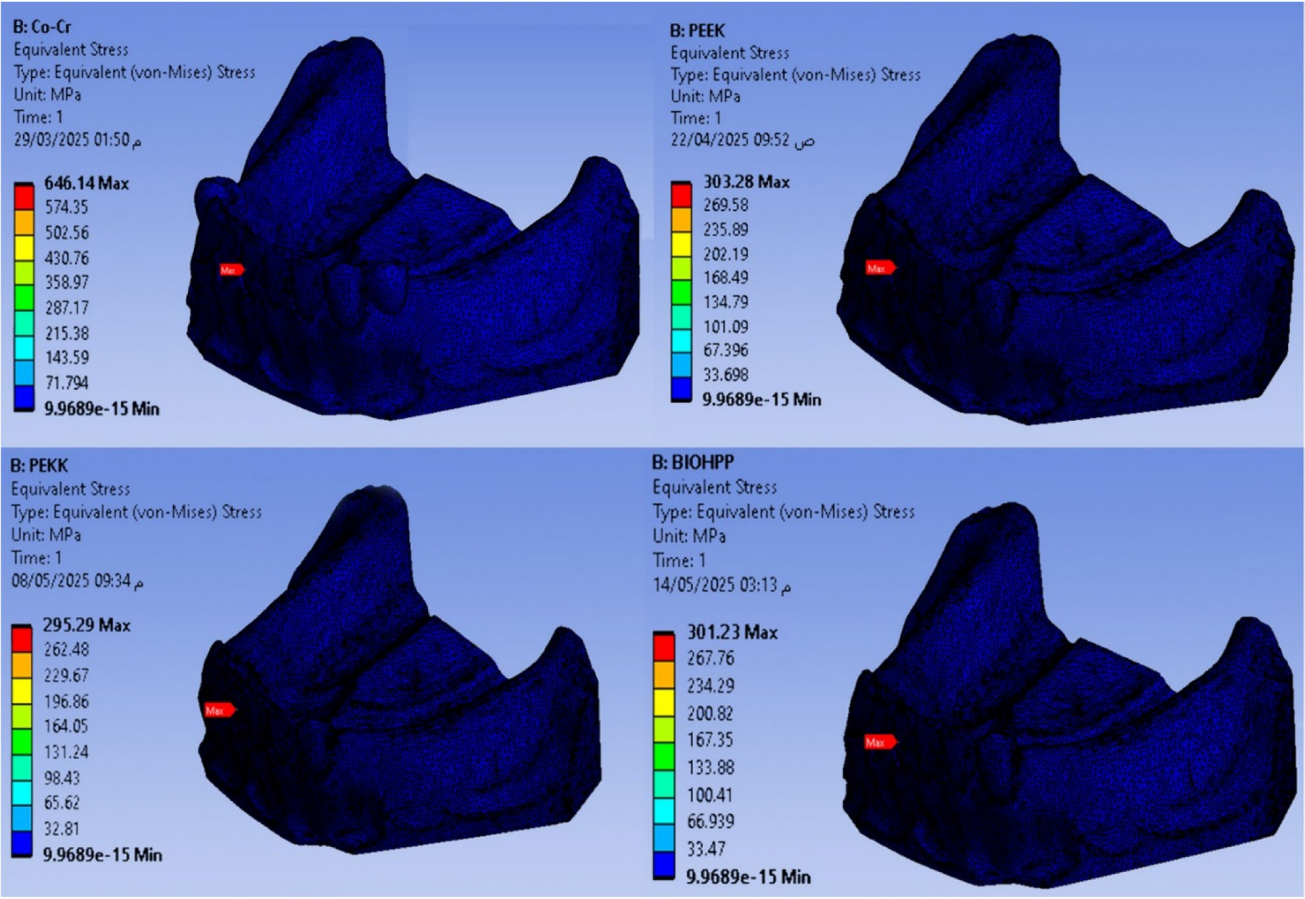
Von misses stress simulation in alveolar bone

**Fig. 6:**
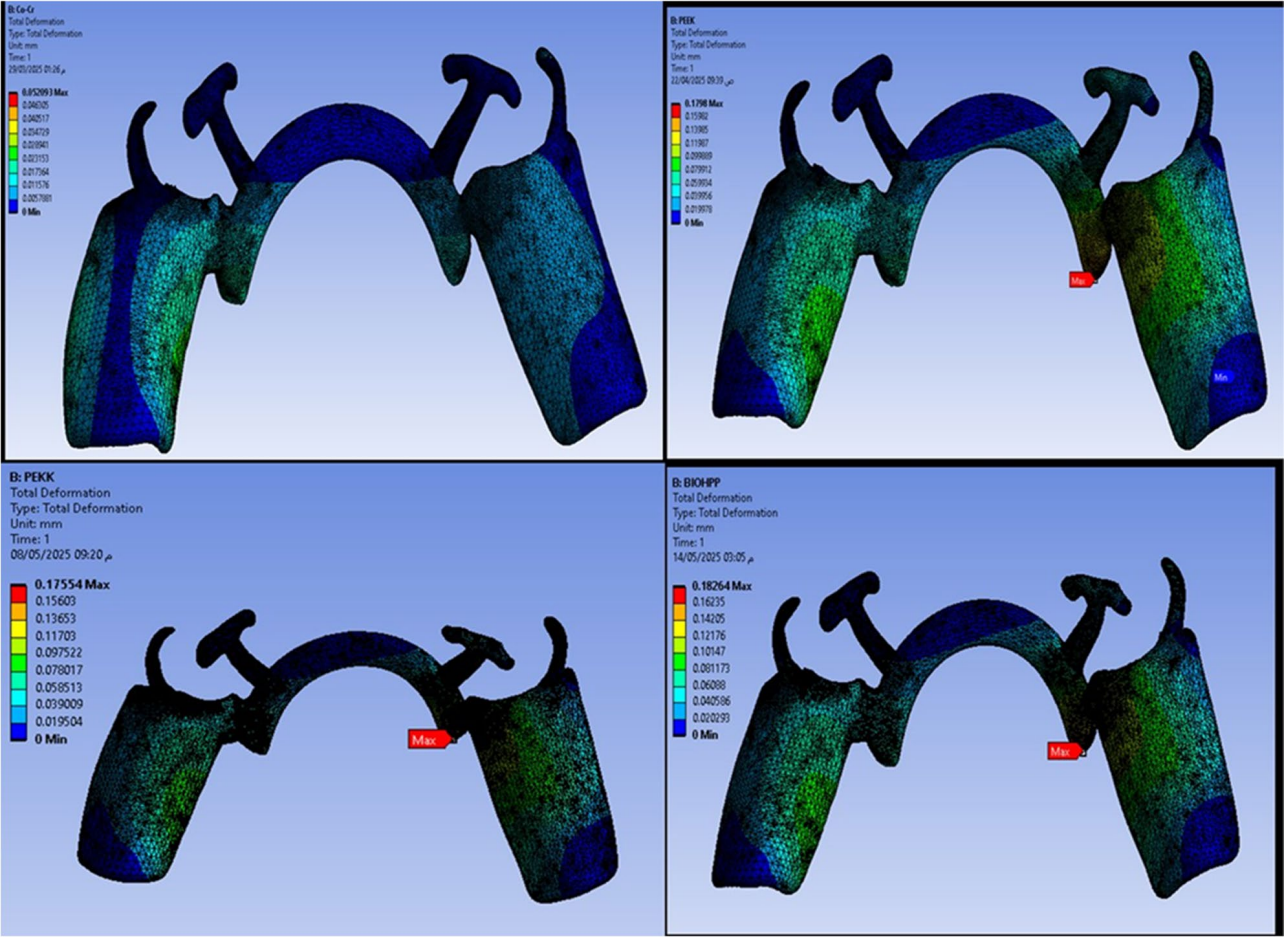
Deformation analysis in metal connector

 Maximum and minimum von Mises stress (MPa) are shown in Figs. 2, 3, 4, 5, and Total deformation (mm) in Fig. 6 in the metal framework, including the connector, abutment teeth, mucosa, and alveolar bone, respectively.

The mandibular model was constructed using SolidWorks 2023 based on standard anatomical dimensions. The alveolar bone was modeled with 1 mm cortical and 2 mm cancellous layers, and a 2 mm mucosal layer was added above the residual ridge. Artificial teeth and denture base geometries were created in CAD following prosthodontic design standards.

Three-dimensional mandibular models were created in SolidWorks 2023 (Dassault Systems, France) based on standard anatomical dimensions. The alveolar bone was modeled with 1 mm cortical and 2 mm cancellous layers, and a 2 mm mucosal layer was added above the residual ridge. Artificial teeth and denture base geometries were created in CAD following prosthodontic design standards and analyzed using ANSYS Workbench 2019 R3 (ANSYS Inc., USA). The same meshing strategy, element type, and boundary conditions were applied to all materials to ensure reproducible and comparable results.

Finite-element analysis produces deterministic numerical outputs; therefore, conventional statistical parameters such as standard deviation are not applicable. Quantitative comparisons were instead based on computed maximum values of von Mises stress and total deformation extracted from identical boundary conditions.

## Result

Post-processing focused on von Mises stress distribution and total deformation, which represent the primary indicators of structural integrity and load transfer in finite-element evaluation of dental frameworks (Chen et al., [17]; Jantaban et al., [38]). These parameters were selected to ensure direct comparability among framework materials. Fatigue-life or durability predictions were not included because such analyses require empirical cyclic-load data that were beyond the scope of this comparative study.

**Fig. 7:**
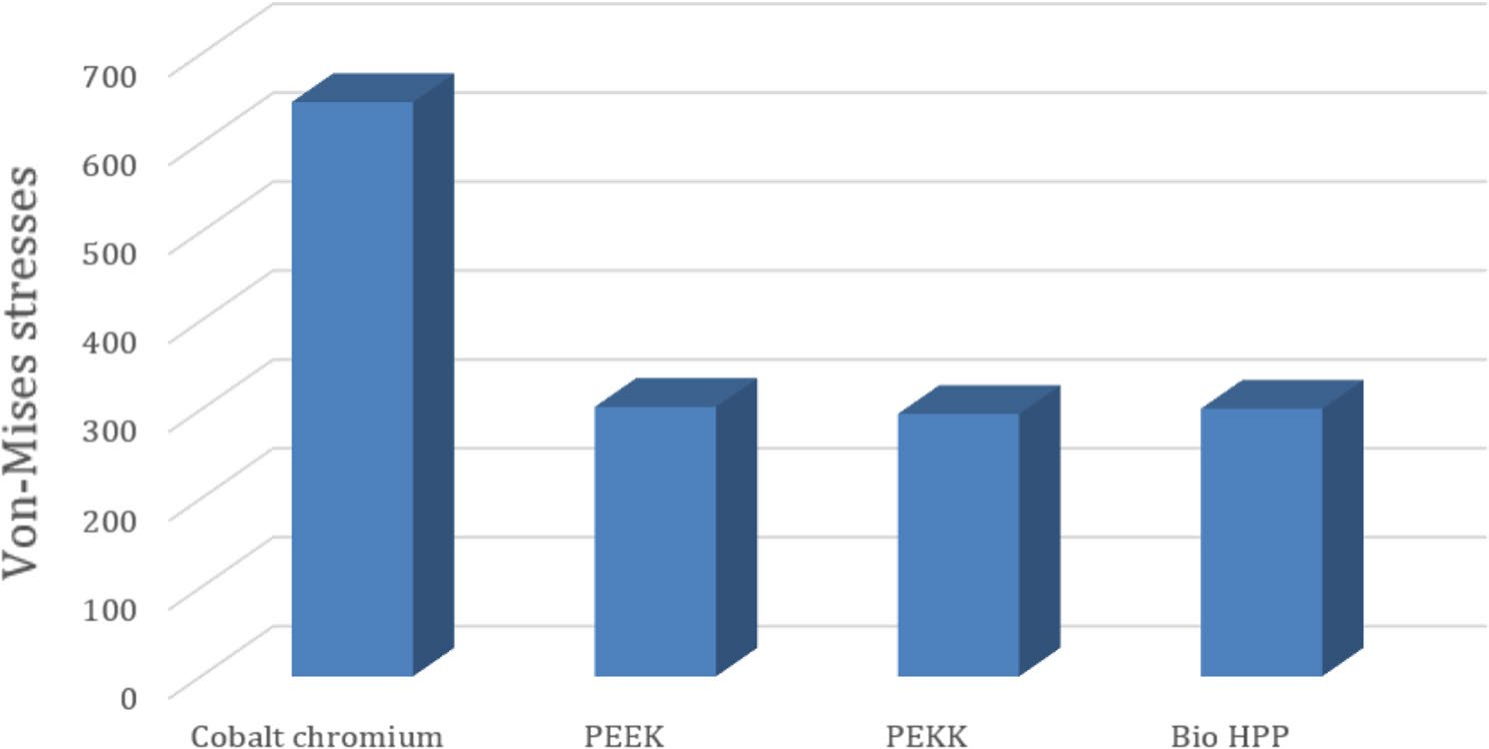
Total framework with lingual bar major connector (Maximum Von-Mises stress in MPa)

Table 2 and Fig. 7 revealed that the maximum Von-Mises stress for the framework including the lingual bar was 646.14 MPa, while that for the PEEK was 303.28 MPa. It was for the PEKK about 295.29 MPa, and for Bio HPP was 301.23 MPa. The stress was higher for the cobalt chromium in comparison to that for either PEEK, PEKK, or Bio HPP.

**Table 2.**
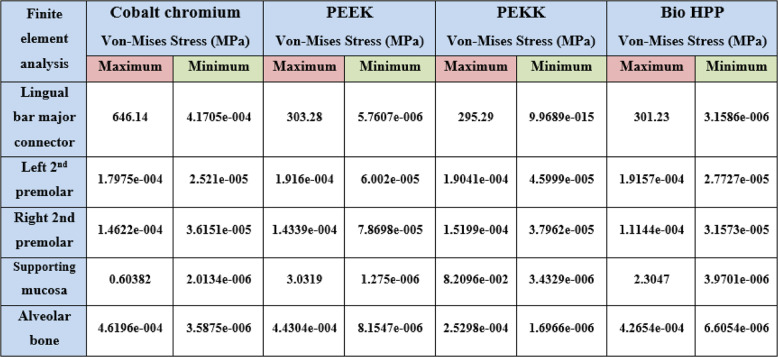
The maximum and minimum values of von Mises stress (MPa) for the mandibular class I RPD constructed with a cobalt chromium, PEEK, PEKK, and BioHPP lingual bar (50x5x3)

**Fig. 8:**
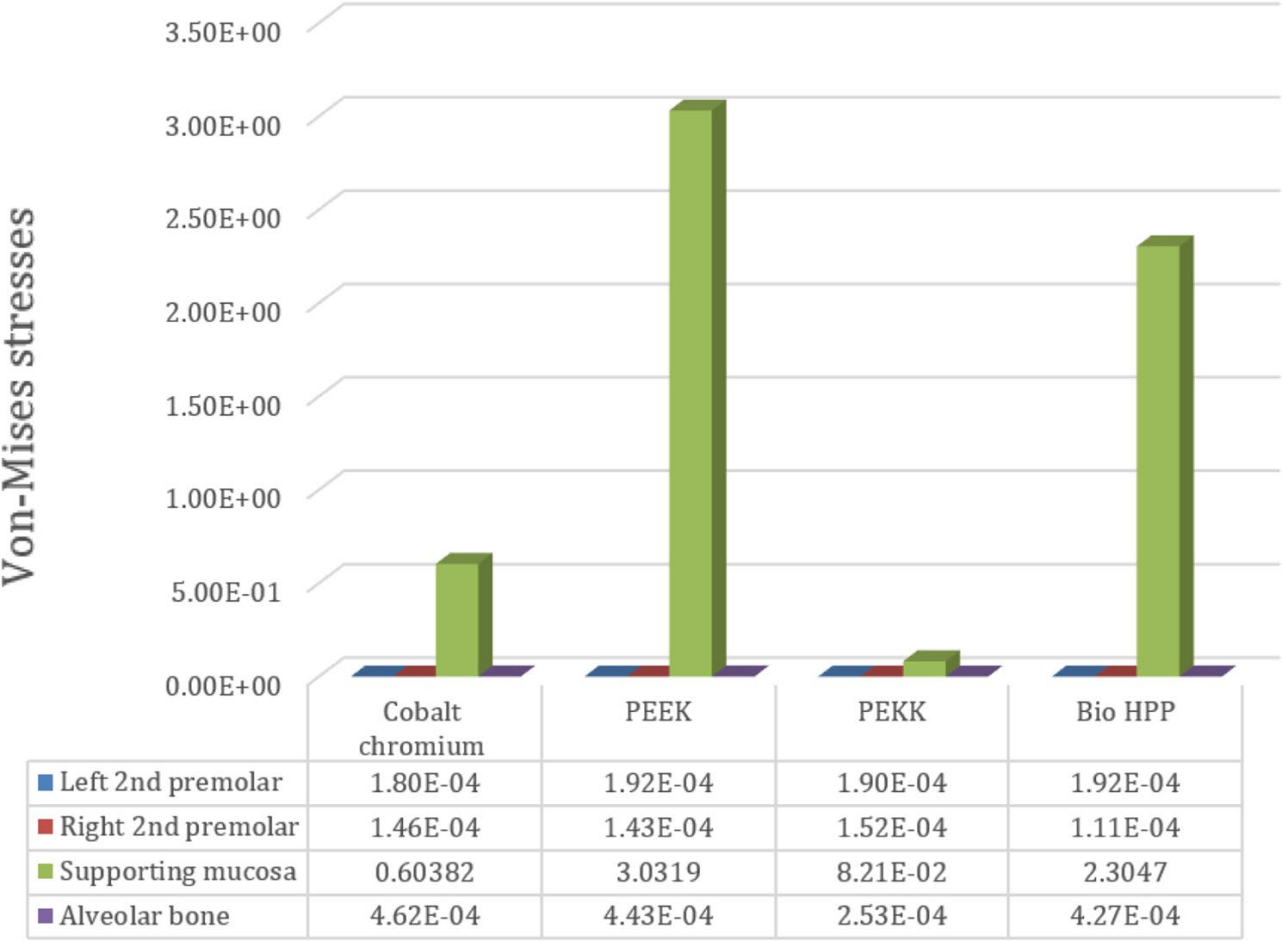
Von-mises stress (MPa) applied on supporting structures of the RPD with different frameworks. material

**Fig. 9:**
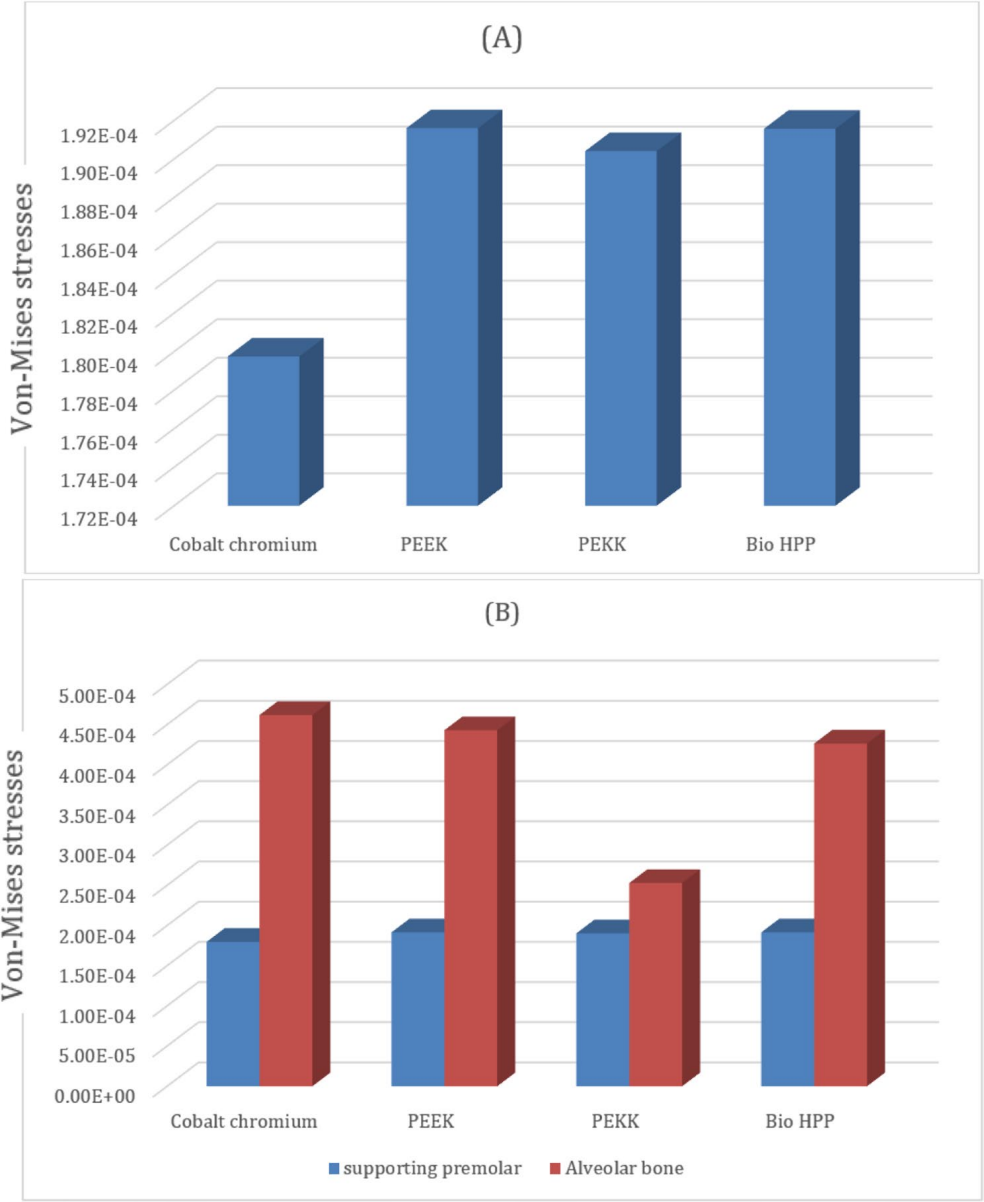
Metal framework with different material (Maximum Von-Mises stress in MPa) applied on supporting teeth (**A**), Metal framework with Lingual bar major connector (Maximum Von-Mises stress in MPa) applied on the supporting premolar and Alveolar bone (**B**)

Comparing the maximum Von Mises stress for the supporting structures of the RPD revealed that higher stress was transmitted to the supporting mucosa, whether with Co-Cr, PEEK, PEKK, or Bio HPP, than to the stresses transmitted to the abutment teeth or alveolar bone. The analysis of stresses transmitted to mucosa showed that PEKK transmits the least stress, followed by cobalt chromium, Bio HPP, and then PEEK. Comparing the stresses transmitted to the abutment teeth and alveolar bone revealed that, regardless of the material used, the stresses transmitted to the bone were greater than those transmitted to the supporting teeth. Cobalt chromium transmits less stress to the supporting teeth, while PEKK transmits the least stress to the bone as shown in Figs. 8 and 9.

**Fig. 10:**
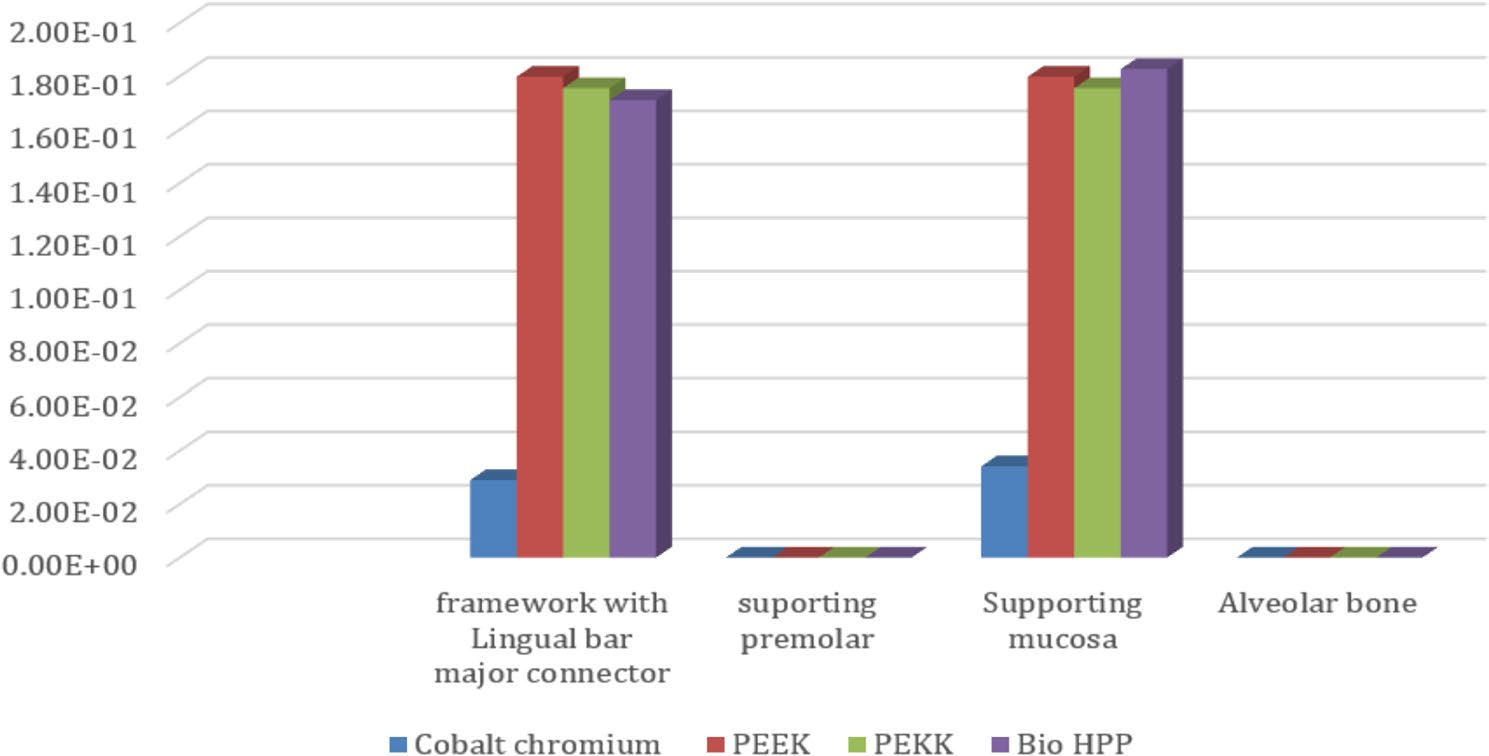
Deformation in mm of framework with different materials

**Fig. 11:**
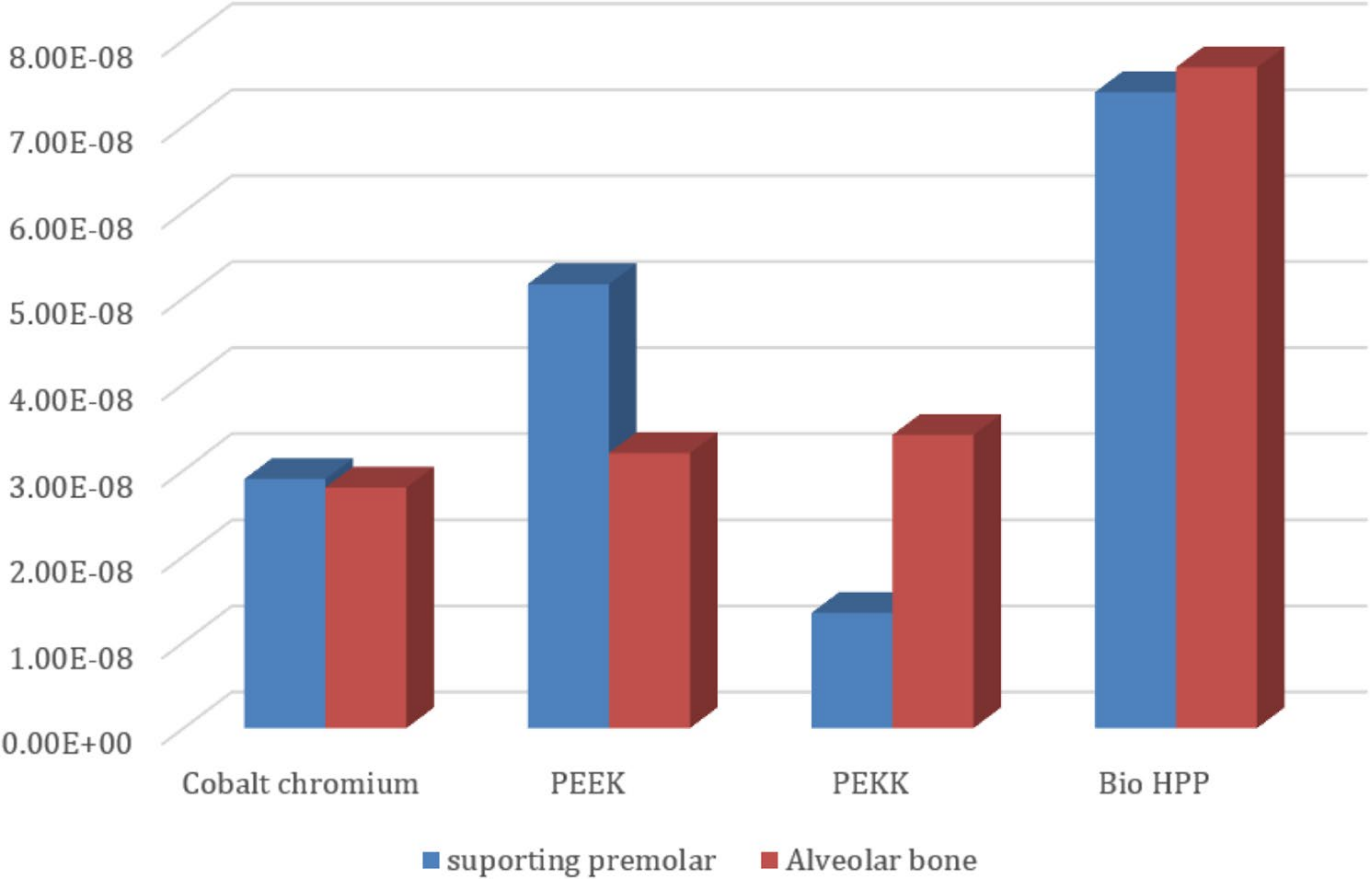
Deformation in mm of supporting premolar and alveolar bone with different materials

On the other hand, Table 3 and Fig. 10 revealed that the maximum deformation was higher for the PEEK, PEKK, and Bio HPP frameworks, where it was 0.1798, 0.17554, and 0.17104 mm, respectively, in comparison to that for cobalt chromium, where it was 0.028999 mm. Also, higher maximum deformations were observed for supporting mucosa either in the case of Co-Cr, PEEK, PEKK, or Bio HPP. Alveolar bone and supporting teeth received less stress and experienced less deformation as shown in Fig. 11. The least deformation of the supporting premolar was observed in the case of the PEKK Framework, but the least deformation of the bone was observed in the case of cobalt chromium framework.

**Table 3.**
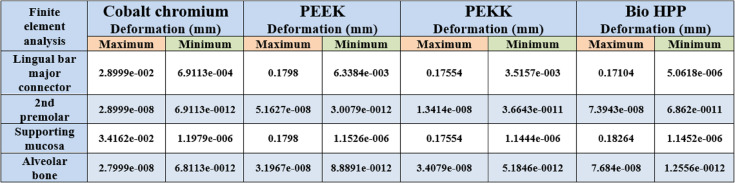
The maximum, minimum values of deformation (mm) for the mandibular class I RPD constructed with a cobalt chromium, PEEK, PEKK, and BioHPP lingual bar (50x5x3)

## Discussion

The present 3D finite element analysis evaluated the biomechanical behavior of mandibular Kennedy Class I removable partial dentures (RPDs) constructed using four different framework materials: cobalt-chromium (Co-Cr), PEEK, PEKK, and BioHPP. A mandibular Kennedy Class I arch was selected as the clinical model due to its reliance on dual support, teeth, and mucosal support, and susceptibility to stress-related complications. The 3D FE is an efficient and reliable method for determining stress and strain distributions in various oral structures [49–53]. It was used in this study to evaluate the behavior of these materials when utilized as a framework for RPDs. The results highlighted the clinical significance of material selection in RPD design, revealing significant variations in the materials’ stress distribution and deformation patterns.

It was demonstrated that the 3D model produced by FEA could accurately replicate the relative material displacements and settling of the soft tissues that support them under various loads and movements. A reliable method for assessing performance and comparing the different materials and design components of removable partial dental prostheses is mathematical modeling simulation of linear viscoelastic soft tissue responses using 3D finite element analysis [48]. Furthermore, advances in tools like ANSYS© and SolidWorks© Simulation have improved the accuracy of stress mapping in denture components, which were used.

In this investigation, five vertical load application spots of 40 N testing loads were placed along the occlusal surfaces of the premolars and molars for a total applied load of 200 N. This was done in accordance with Nakamura et al. [48], in addition to an oblique load of 150 N that was applied bilaterally on the first and second molars to simulate an occlusal force.

Lin et al. [54] demonstrated how changes in the material properties of framework components directly impact stress concentrations on abutment teeth and residual ridges. This comes in agreement with the study results, where each material used for the RPD metal framework differs in stress amount and concentration.

There is a biomechanical rationale connecting Young’s modulus, stress transfer, and major connector deflection. Studies on how material flexibility affects stress transmission to supporting structures include Satheesh Kumar et al. [26], who directly analyze rigidity and deflection in major connectors and show how materials with lower modulus lead to greater deformation. Chen et al. [17] illustrate how different elastic moduli impact rigidity, stress transfer, and mucosal loading in RPDs.

Cobalt-chromium (Co-Cr) alloys have traditionally been the material of choice for constructing RPD frameworks due to their high strength, rigidity, and corrosion resistance. With a Young’s modulus of approximately 200 GPa. Co-Cr provides excellent rigidity, allowing thin but durable components, such as lingual bars, to be fabricated [55]. However, the rigidity of Co-Cr can be a double-edged sword. While it minimizes deformation, it can result in high stress concentration at the osseous support structures.

The present FEA findings correspond closely with experimental data reported in previous investigations. Fahmy et al. [56] demonstrated through strain gauge analysis that the polyetherketoneketone material causes less stress on the remaining ridges and stress on the abutments than the cobalt-chromium ones. It might therefore be advised for RPDs that are supported by weak abutments, a pattern also observed in our simulations. Similarly, Amaral et al. [33] reported comparable Photoelastic FEA stress trends for PEEK-based dentures. The coherence between our simulated stress distribution and these validated studies supports the reliability of the current numerical model despite the absence of direct in vitro testing.

Jantaban et al. [38] found that the Co-Cr framework exhibited stress levels up to four times higher than those in the PEEK framework, due to differences in their mechanical properties. Co-Cr, with a high Young’s modulus (200–220 GPa) is highly rigid and concentrates stress, while PEEK, with a lower modulus (3–4 GPa), offered greater flexibility and distributed stress more evenly. These findings agree with those of Hussein et al. [24], who also stated that PEEK shows reduced stress in the framework while retaining enough strength for clinical application.

In this study, Co-Cr framework demonstrated the highest Von-Mises stress value, likely due to its high elastic modulus and stiffness, leading to greater resistance to deformation and consequently higher stress concentrations under load [57, 58]. In contrast, the polymer-based materials PEEK, PEKK, and BioHPP demonstrated significantly lower stress values, consistent with their lower elastic moduli and ability to distribute occlusal forces more uniformly, and reducing the peak stress experienced by the framework and supporting structures [59–61]. Because the design of the model matches the mechanical model in terms of shape, dimension, and location of each RPD component, the simulation in this study matches the mechanical tests. The Von-Miss Stress applied to the mucosa was found to be 0.6 in the 3D FEA utilizing the Co-Cr metal framework, which matches the 0.59 finding in the earlier studies [17].

However, this stress relief comes at the cost of increased deformation. PEEK exhibited the highest deformation, followed closely by PEKK and BioHPP. This behavior may be related to the fact that the elastic modulus of PEEK material is closer to PEKK and BioHPP compared to other framework materials. Whereas Co-Cr framework showed minimal deformation. These results are in line with previous finite element analyses [62], which reported that materials having a lower Young’s modulus cause greater stress on the supporting structure and deflection. In polymer-based materials showed the largest displacement compared with Co-Cr. As a framework material, standard rigid metal alloys were found to be more advantageous than polymer materials in terms of stress distribution.

The results of this study suggest that denture stability during function may be compromised by the comparatively greater deformation seen with PEEK and BioHPP components. Overly flexible major connectors may allow unintended rotational movements of the prosthesis, which, over time, may cause higher mucosal loading, decreased retention, and even discomfort for the patient [17]. Compared to cobalt-chromium showed reduced deformation values while still transferring less stress to the mucosa and abutment teeth. PEKK may provide a better compromise between stiffness and flexibility, preventing high stress concentrations on the supporting tissues while offering enough resistance to connection bending. Increased clinical performance and patient comfort in mandibular Class I RPDs could result from such a biomechanical profile [63]. 

Concerning stress transmission to supporting tissues in this finite analysis study, the Previous biomechanical research, such as that conducted by Kumar et al. in 2021, examined the impact of class I RPD on the mucosa and discovered that the highest stresses were 6 MPa. Additionally, they investigated the impact of this prosthesis on patient satisfaction and discovered that there were no negative effects. Additionally, they discovered that the flexible denture foundation offered excellent patient satisfaction while causing a maximum stress of 25 MPa [64]. In this study, the displacement of the free end of the PEEK, and Bio HPP framework were larger than cobalt chromium, which was a disadvantage regarding the stability of the denture and increased the stresses to the supporting mucosa. This result agrees with Chen et al.‘s finite element analysis [17].

Within the constraints of static loading and idealized boundary conditions, PEKK demonstrated the most balanced combination of rigidity and flexibility, yielding lower mucosal stress and moderate framework deformation. Its biomaterials display ultra-high performance among all thermoplastic composites for exceptional mechanical strength, chemical resistance, and great thermal stability. They are also an elastic material with good shock absorbance and fracture resistance [65]. According to a study by Stawarczyk B. et al. [59] PEKK has a suitable degree of fracture resistance, and its capacity to disperse stress and absorb shock suggests that it might be developed into a novel restorative material that can take the place of metals and ceramics [66]. PEKK’s strength (65 MPa), fracture resistance, and capacity to absorb stress all increase the material’s potential for use as a restorative. In contrast to dentin, the PEKK has a lower modulus of elasticity yet a comparable compression strength [67]. PEKK may be regarded as a thermoplastic polymeric substance with shock-absorbing capabilities, but when it is aged by thermocycling, it deforms and loses strength and flexural modulus. Additionally, it puts additional strain on the supporting structures [68]. The importance of thermal cycling and oral fluid exposure in altering mechanical properties, suggesting that clinical performance may differ from idealized in-vitro or simulated conditions.

In this current study of a Class I RPD, 3D FEA, the PEKK framework provides the lowest transfer of stresses to the supporting structures of teeth, mucosa and bone. This are confirmed by mechanical reasoning. PEKK’s improved energy absorption and damping (through its microstructural characteristics), better fatigue resilience, more favorable deformation compatibility with adjacent components, and balancing of stiffness are the major factors. PEKK can function better as a “stress buffer” under functional loading than PEEK (slightly more yielding, lower stiffness) and BioHPP (stiffer, more brittle composite) [69–71]. 

In summary, while Co-Cr frameworks provide superior rigidity and minimal deformation, high-performance polymers, especially PEKK, offer favorable stress distribution and tissue preservation. These findings suggest that PEKK may be a promising alternative for metal-free RPD frameworks, particularly in cases requiring optimal load dissipation and aesthetic considerations. This finding supports the idea that material selection in RPD design should consider not only mechanical properties but also the biological implications for the oral environment. Clinical trials are needed to assess the long-term performance of mandibular class I with PEEK, PEKK, and BioHPP, RPD prostheses.

This study has inherent limitations typical of finite element analysis. Linear elastic assumptions and an idealized mandibular Class I model were used, without accounting for viscoelastic or fatigue effects. Although experimental validation was not included, mesh convergence and validated protocols were ensured. Future work will incorporate nonlinear behavior, cyclic loading, and patient-specific geometries to enhance clinical relevance.

Despite these limitations, comparative analysis offers valuable insights into the mechanical performance of various framework materials.

## Conclusion

Using the same size of PEEK and Bio-HPP as the ideal dimensions of cobalt chromium major connector of the mandibular class I RPD provides more stress and deflection to the supporting structure, teeth, and soft tissues. This can be attributed to the less rigidity of PEEK and Bio-HPP in comparison to the cobalt chromium. On the other hand, among the tested materials, PEKK exhibited the most favorable balance between stiffness and flexibility under the simulated conditions. Further research incorporating cyclic-fatigue and wear testing is required before drawing definitive conclusions regarding material superiority in clinical performance.

### The practical implications of these findings

PEKK is considered the most suitable non-metallic material for construction RPD frameworks because it protects the mucosa, alveolar bone, and abutment teeth from excessive stress. In addition to that, PEKK offers more printable polymers than PEEK in terms of digital manufacturing [67].

## Supplementary Information

Supplementary Material 1.

Supplementary Material 2.

## Data Availability

The datasets used in the current study are available from the corresponding author upon request.

## Abbreviations

3D: Three-dimensional
FEA: Finite Element Analysis
RPD: Removable Partial Denture
PEEK: Polyetheretherketone
PEKK: Polyetherketoneketone
BioHPP: Biocompatible High-Performance Polymer
Co-Cr: Cobalt-Chromium
